# The Effect of Isosorbide Content on the Thermal and Compressive Properties of Closed-Cell Rigid Polyurethane Foam

**DOI:** 10.3390/polym17040495

**Published:** 2025-02-13

**Authors:** Se-Ra Shin, Dai-Soo Lee

**Affiliations:** 1 Research Institute, Jungwoo Fine Co., Ltd., #63-8, 75, Seogam-ro 1-gil, Iksan-si 54586, Jeonbuk-do, Republic of Korea; 2 C & S Partner, B-402, Hanam Technovalley U1 Center, Hanam-si 12982, Gyeonggi-do, Republic of Korea; dslee@jbnu.ac.kr

**Keywords:** isosorbide, rigid polyurethane foam, closed-cell foam, thermal insulation properties, mechanical properties, reversibility

## Abstract

In this study, isosorbide (ISB), a bio-based compound derived from the dehydration of D-sorbitol, was used as a sustainable resource to produce various types of rigid polyurethane foams (RPUFs). Featuring a rigid bicyclic structure and two hydroxyl groups, ISB was solubilized in polyether polyol at concentrations up to 10 wt% for RPUF production. ISB-based RPUFs exhibited smaller and more uniform cell sizes, along with a 4% increase in closed-cell content, resulting in improved thermal insulation with a 4.69% reduction in thermal conductivity. Additionally, the compressive strength increased by up to 31%, enhancing the material’s durability. The compatibility of ISB with conventional raw materials and its unique structure contributed to these improvements. The reversible urethane bond formation of ISB was also observed, influencing cell structure during foaming. These results indicate that ISB can effectively improve both the thermal insulation properties and material durability under compression of RPUFs, making it a promising material for sustainable applications.

## 1. Introduction

Polyurethane foam (PUF) is a polymer with diverse chemical structure, primarily produced through the reaction of hydroxyl functional groups in polyol and isocyanates. PUF is widely utilized in various industrial fields, such as construction, furniture, packaging, and medical devices, due to its excellent mechanical properties and durability. PUF can be classified into rigid and flexible foams based on cell morphology and characteristics. Rigid polyurethane foam (RPUF) with a closed-cellular structure has low thermal conductivity, making it suitable for use as insulation materials in construction, pipes, and refrigerators [1,2,3,4,5,6]. In contrast, flexible polyurethane foam with an open-cellular structure, exhibiting superior elasticity and air permeability, is widely used in furniture, mattresses, and cushioning materials. The low thermal conductivity of RPUF is a crucial property for the insulation property, influenced by several factors, including density, cell size, closed-cell content, and the thermal conductivity of the gas trapped in the foam cells [2,4,7,8]. The thermal conductivity of RPUF is typically the sum of contributions from the solid phase ($k_{s}$), the gas phase ($k_{g}$), and radiation across the cells ($k_{r}$). While the thermal conductivities of the solid and gas phases are constant because the raw materials are fixed, improving the insulation properties of RPUFs often involves minimizing radiative heat transfer ($k_{r}$), which can be achieved by reducing cell size or incorporating nanofillers [9,10,11,12,13,14].

Recently, with the increased concern for environmental issues, many studies have been focused on replacing petroleum-based raw materials with bio-based alternatives. Many studies have investigated the use or modification of plant-oil-based bio-raw materials to obtain bio-polyols for RPUF [15,16,17,18,19,20,21,22]. P. Acuna et al. [15] reported on the preparation of an RPUF composite using castor oil modified with diethanolamine and phenylphosphonic acid, which resulted in enhanced insulation, mechanical strength, and flame retardancy. However, the thermal conductivity of bio-based RPUF was found to be higher than that of RPUF produced with conventional polyol. Borowicz et al. [16] reported the synthesis of rigid polyurethane/isocyanurate foam using mustard seed oil modified with 2,2${\,}^{\prime }$-thioethanol, achieving lower thermal conductivity and superior flame retardancy compared to petroleum-based RPUF. The flexibility of the long aliphatic chains in bio-polyols has been observed to lead to an increase in cell size and a decrease in mechanical strength with increased bio-polyol content, which caused increased thermal diffusion across the cell and reduced thermal conductivity. Zhang et al. [17] synthesized RPUF using a soy-castor oil-based polyol, noting a decrease in cell size and an increase in thermal stability with increasing bio-polyol content, although compressive strength and insulation properties were decreased. While plant oil-based bio-polyols offer promising advantages, their high viscosity and large molecular weight may result in trade-offs in key properties, such as thermal conductivity and material durability, compared to control group. To address this, we began searching for a complementary material and considered ISB as a potential solution in this case.

Isosorbide (ISB), a bio-based compound derived from the dehydration of D-sorbitol, has emerged as a promising alternative to petroleum-based resources [23,24,25,26,27,28,29,30,31,32,33]. ISB is renewable, biodegradable, non-toxic, and biocompatible, making it an attractive candidate for sustainable polymer applications. Its rigid bicyclic structure, consisting of two tetrahydrofuran rings and reactive hydroxyl groups at carbons 2 (exo) and 5 (endo), provides unique advantages in enhancing thermal stability, mechanical strength, and ultraviolet resistance in polymers [34,35]. These properties make ISB a viable substitute for petrochemical-based compounds, such as bisphenol-A, and it has been applied in industries ranging from food packaging to medical devices and coating [32,36,37,38,39]. Given ISB’s structural similarity to phenolic hydroxyl groups, which are known to reversibly form urethane bonds with isocyanates, we expect that the hydroxyl groups in ISB can also react reversibly with isocyanates to form urethane units that dissociate at specific temperature [40,41,42,43,44].

In this study, ISB formed a homogeneous solution in RPUF as a bio-based resource through dissolution in conventional polyether polyol. The effects of ISB on thermal and mechanical properties of RPUF were investigated in relation to the reversibility of urethane units formed by the reaction of hydroxyl groups in ISB with isocyanates. The results indicates that the incorporation of ISB led to a smaller and more uniform cell structure, thereby enhancing the thermal insulation properties of RPUF without significantly compromising their mechanical strength.

## 2. Materials and Methods

### 2.1. Materials

ISB (146.1 g/mol of molecular weight) was obtained from Samyang Co., Ltd. (Daejeon, Republic of Korea). A conventional polyether-type polyol based on sugar/glycerin with a hydroxyl number of 430 mgKOH/g (JOP-0655) was provided by Jung Woo Fine Chem Co., Ltd. (Iksan, Republic of Korea). Polymeric 4,4-diphenylmethane diisocyanate (pMDI, Cosmate M200) was obtained from Kumho Mitsui Chemicals (Yeosu, Republic of Korea). Dimethyl-cyclohexylamine (Polycat^®^ 8, PC-8) and pentamethyldiethylenetriamine (Polycat^®^ 5, PC-5) from AirProducts and Chemicals (Allentown, PA, USA) were used as foaming catalysts. Silicon surfactant (B-8462) and tris(1-chloro-2-propyl) phosphate (TCPP) were obtained from Evonik (Essen, Germany) and Sigma-Aldrich (Yongin, Republic of Korea), respectively. SOLKANE^®^ 365/227 from Solvay (Brussels, Belgium) and distilled water were used as blowing agents. JOP-0655 was dehydrated at 70 ${\,}^{\circ }C$ for 24 h under vacuum prior to use. All chemicals were used as received.

### 2.2. Preparation of RPUFs Based on ISB

**Figure 1:**
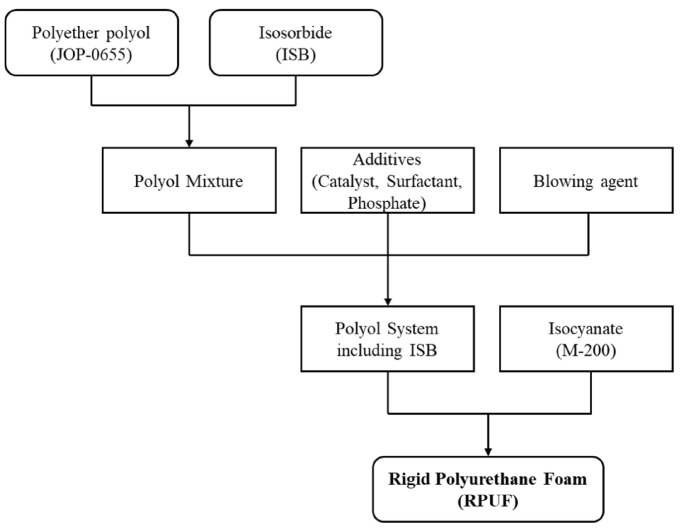
Procedure for the preparation of ISB-based RPUFs.

**Figure 2:**
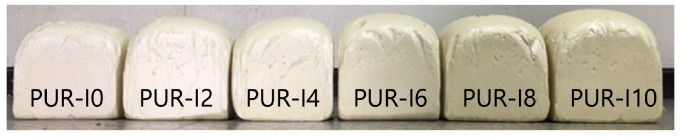
Photographs of manufactured RPUFs.

Following the procedure in Figure 1, different amounts of ISB (0, 2, 4, 6, 8, and 10 wt% relative to the total polyol weight) were dissolved in JOP-0655 at 90 ${\,}^{\circ }C$ to create transparent ISB-polyol mixtures. The ISB-polyol mixtures were then cooled to room temperature, and no visible precipitates or crystals were observed after cooling. RPUFs with different ISB contents were prepared in two steps. First, predetermined amounts of surfactant, catalyst, phosphate, and blowing agents were added to the polyol and homogeneously mixed under mechanical stirring. Second, the stoichiometrically required amount of pMDI was added to the mixture prepared in the first step, and the mixture was vigorously stirred at 6000 rpm for 7 s. The resulting mixture was quickly poured into a steel mold (300 mm × 300 mm × 50 mm) with a lid. After curing at 60 ${\,}^{\circ }C$ for 20 min, the RPUFs were demolded and stored at room temperature for at least 24 h before characterization. The RPUFs with different ISB contents were designed to have similar densities of 45 kg/$m^{3}$. The NCO index was maintained at a constant value of 120. Each foam was manufactured at least three times, and the processing parameters for each were recorded and averaged. Table 1 summarizes the formulations of the RPUFs with different recycled polyol contents, while Figure 2 presents the corresponding RPUFs images.

### 2.3. Characterization

To characterize the reactivity of the RPUFs on foaming and polymerization, the cream time, gel time, and tack-free time were determined using a stopwatch according to ASTM D7487-13 [45].

The cell morphology of the RPUFs was studied using a scanning electron microscope (AIS2100C, SERON Technologies, Seoul, Republic of Korea) at an accelerating voltage of 20 kV. The samples were cut using a razor blade and coated with gold before observation to avoid electron charging. The number of cells per unit ${\mathrm{cm}}^{3}$ was calculated using following equation [46]:

(1) $$N_{f}\left({\mathrm{cells}/\mathrm{cm}}^{3}\right)={\left({\mathrm{nM}}^{2}/A\right)}^{3/2}$$

where $N_{f}$ is the number of cells per ${\mathrm{cm}}^{3}$, n is the number of cells in the micrograph, M is the magnification factor of the micrograph, and A is the area of the micrograph (${\mathrm{cm}}^{2}$).

The closed cell contents of the samples were measured with an ULTRAPYC 1200 instrument from Quantachrome (Boynton Beach, FL, USA), following ASTM D 6226 [47] with sample dimensions of 25 × 25 × 25 ${\mathrm{mm}}^{3}$. The closed cell contents of five specimens per sample were measured and averaged.

The thermal conductivities of the RPUF were obtained by using a heat-flow meter (HC-074, EKO, Tokyo, Japan) with the two plane plates maintained at different temperatures according to ASTM C 518 [48]. The thermal conductivities of three specimens per sample were measured, and the average values were obtained.

Regeneration of NCO functional groups by a thermo-reversible feature was monitored using Fourier-transform infrared (FTIR) spectrometry (JASCO FTIR 2000, Easton, MD, USA) with a heating block, thermocouple, and digital thermal controller. FTIR spectra were collected in transmittance mode in the wavenumber range $4000\;\mathrm{to}\;500\;{\mathrm{cm}}^{-1}$ at a resolution of $4\;{\mathrm{cm}}^{-1}$.

The dynamic mechanical properties of the RPUFs were studied using dynamic mechanical analysis (DMA, Q800, TA Instruments, New Castle, DE, USA). The tests were performed in tension mode from 30 ${\,}^{\circ }C$ to 250 ${\,}^{\circ }C$ at a heating rate of 5 ${\,}^{\circ }C$/min (frequency 1 Hz and amplitude 15%). Stress relaxation behaviors were investigated at 160 ${\,}^{\circ }C$ at 1% strain.

The compressive strengths in both the blowing direction and that perpendicular to blowing of the RPUFs were measured using a universal testing machine according to ASTM D 1621 [49] with a sample size of 40 × 40 × 40 ${\mathrm{mm}}^{3}$. The crosshead speed was 2 mm/s, and five specimens per sample were measured and averaged. To eliminate the effect of density, the compressive strengths measured were normalized for both the blowing and perpendicular directions according to the following equation [3]:

(2) $$\sigma_{n}=\sigma {\left(\frac{40}{\rho }\right)}^{2}{\left(\frac{1+\sqrt{40/\rho_{s}}}{1+\sqrt{\rho /\rho_{s}}}\right)}^{2}$$

where $\sigma_{n}$ is the normalized compressive strength, σ is the experimental compressive strength, ρ is the density of the RPUF (kg/$m^{3}$), and $\rho_{s}$ is the density of solid polyurethane (1200 kg/$m^{3}$).

The thermal decomposition behavior of RPUFS was investigated by thermogravimetric analysis (TGA, Q50, TA Instruments, New Castle, DE, USA). About 5 mg sample was loaded on the platinum pan, and thermogravimetry (TG) and derivative TG curves were obtained from 30 ${\,}^{\circ }C$ to 800 ${\,}^{\circ }C$ at a heating rate of 20 ${\,}^{\circ }C$/min under nitrogen atmosphere.

The water absorption percentage was determined according to ASTM D570-98 [50]. Rectangular samples with a size of 40 × 40 × 10 ${\mathrm{mm}}^{3}$ were immersed in distilled water at 25 ${\,}^{\circ }C$. After 24 h, the specimens were wiped to remove the surface water and weighed. The percentage weight increase after measurement was calculated.

## 3. Results

Although the changes in the overall shape and color of RPUFs according to the ISB content can be observed in Figure 2, no significant differences were noted. However, there were variations in the foaming process parameters. The cream time (CT) marks the starting point of bubble formation and foam rise, indicated by the mixture changing from dark yellow to creamy yellow. The gel time (GT) corresponds to the point of gelation and cross-linking through the formation of urethane and urea linkages, while the tack-free time (TFT) is when the foam surface loses its stickiness. The density of RPUFs significantly affects their mechanical properties. The density remained nearly constant, as the blowing conditions were maintained by using the same type and amount of blowing agent. During RPUF preparation, as shown in Table 2, both GT and TFT decreased with increasing ISB content. In contrast, CT remained relatively constant, regardless of the ISB concentration. The increased reactivity of RPUFs containing ISB is attributed to the greater number of hydroxyl groups capable of reacting with isocyanate groups. Additionally, as a short diol, ISB can more easily approach reactive species compared to conventional polyether polyol, which have relatively longer chains. ISB has a low functionality (f=2) compared to other raw materials used in RPUF production, such as polyol(f>4) and pMDI(f≈2.7). This may reduce the cross-linking density of the polymer network, which could lead to non-uniform cell structure and open cells, potentially weakening the cell walls. However, the rigid fused bicyclic structure of ISB was expected to compensate for this reduction in mechanical properties, and its reversible reaction may contribute to enhanced cell formation.

**Figure 3:**
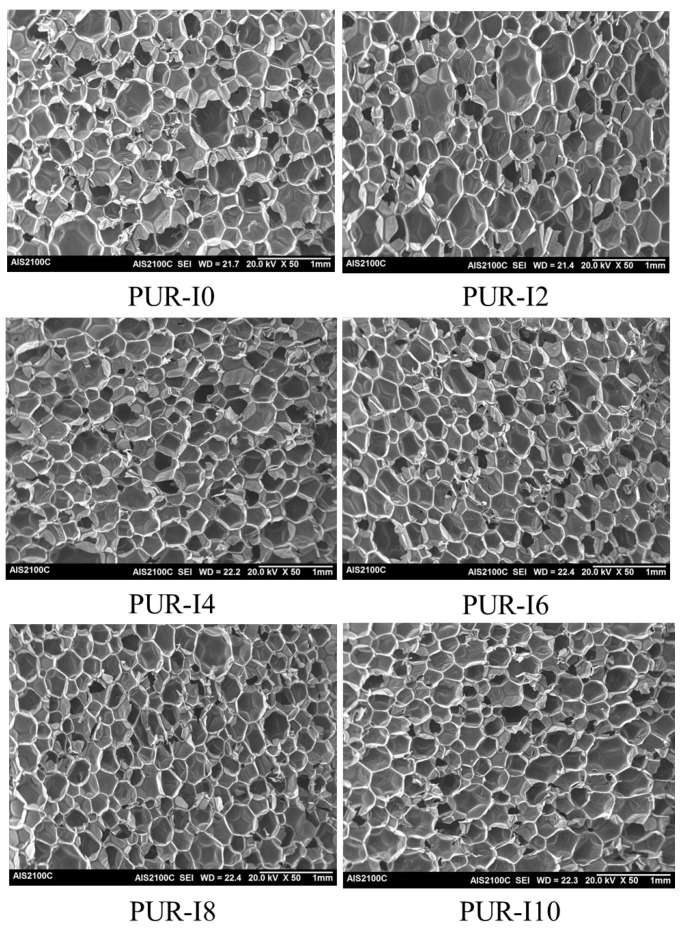
SEM images of RPUFs with various amount of ISB.

Figure 3 shows SEM images of RPUFs with varying ISB contents, and Table 3 summarizes the average cell size, average cell wall thickness, and the number of cells per unit volume (${\mathrm{cm}}^{3}$). All RPUFs exhibited spherical or polyhedral cells without shrinkage or collapse, even at high ISB content. The average cell wall thickness and average cell size decreased from 33.1 μm and 475 μm for PUR-I0 to 22.6 μm and 302 μm for PUR-I10, respectively. Incorporating ISB into RPUFs resulted in a more uniform and homogeneous cell size. Notably, the number of cells per unit volume in PUR-I10 was nearly three times that of PUR-I0. The reduction in average cell size and improved uniformity is attributed to the good compatibility of ISB with the raw materials for RPUFs. ISB, with its hydrophilic and bicyclic structure, has a high affinity for pMDI and distilled water (the blowing agent), improving compatibility and leading to the formation of a finer and more homogeneous cell structure. Another reason for this phenomenon is the decrease in GT and TFT. The increased reactivity may result in a rapid increase in the foam’s modulus, which restricts bubble expansion and growth during foaming.

The reversible properties of ISB-based urethane bonds during the foaming process reduce polymer modulus and impart flexibility to the molecular chains, allowing for a more adaptable response to the blowing pressure caused by the expansion of the blowing gas. However, this can result in a reduction in average cell wall thickness, as shown in Table 3. The decrease in average cell wall thickness may lead to cell rupture or coalescence during the foaming process, ultimately causing a decrease in closed cell content and an increase in average cell size of the resulting RPUFs. Nevertheless, as mentioned earlier, the rapid reactivity of ISB-based RPUFs prevents excessive bubble expansion and growth during foaming, promoting the formation of smaller and more uniform cellular morphology. Additionally, the rigid bicyclic ring structure of ISB enhances the stiffness of the polymer matrix, enabling it to withstand blowing pressure and restrict cell rupture and coalescence occurring due to the cell wall thinning. This, in turn, contributes to an improvement in the strength of the resulting ISB-based RPUFs. Consequently, the reversible properties of ISB-based urethane bonds, combined with the robust structure of the bicyclic ring, promote the formation of smaller and more uniform cells, which leads to enhanced compressive strength and thermal insulation properties of RPUFs.

**Figure 4:**
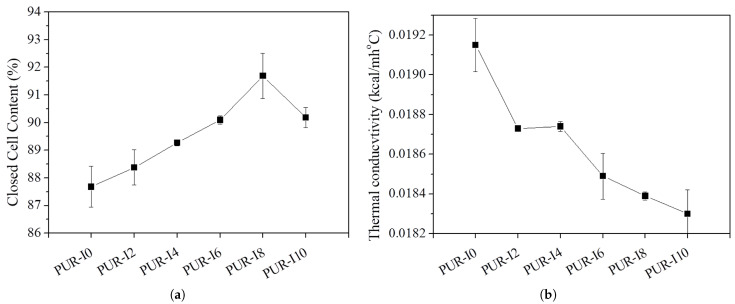
Closed cell content (**a**) and thermal conductivity (**b**) of various RPUFs.

Figure 4 illustrates the closed cell content and thermal conductivity of RPUFs with varying ISB contents. Low thermal conductivity is essential for the application of RPUFs in thermal insulation. Typically, the thermal conductivity of polyurethane foams depends on factors such as average cell size, closed cell content, the thermal conductivity of the blowing gas trapped within the cells, and foam density. In therms of insulation properties, closed cell content is particularly important, as gases such as carbon dioxide 0.0151 kcal/mh ${\,}^{\circ }C$ at 25 ${\,}^{\circ }C$) and HFA-365/227(0.0224 kcal/mh ${\,}^{\circ }C$ at 25 ${\,}^{\circ }C$) are produced during the foaming reaction. When the cells are closed, these gases are trapped inside, helping to maintain low thermal conductivity. The closed cell content of RPUFs increased with the addition of ISB, reaching a maximum of 91.7% for PUR-I8, compared to 87.7% for PUR-I0. Although the closed cell content slightly decreased for RPUFs containing 10 wt% ISB, this decrease in the closed cell content of PUR-I10 is attributed to thinner cell walls caused by a reduced polymer modulus during foaming as ISB content increased. This reduction in wall thickness may lead to cell rupture and the opening of cell windows. Nevertheless, all ISB-based RPUFs exhibited higher closed cell content than the control foam. Additionally, incorporating ISB into RPUFs reduced thermal conductivity, decreasing it from 0.0192 kcal/mh ${\,}^{\circ }C$ for control foam to 0.0183 kcal/mh ${\,}^{\circ }C$ for PUR-I10. Despite the potential drawbacks of increased reactivity and reduced cross-linking density caused by ISB, the closed cell structure was maintained, likely due to the reversible reactions of ISB-based urethane bonds, which contributed to enhanced closed cell content and improved thermal insulation properties.

**Figure 5:**
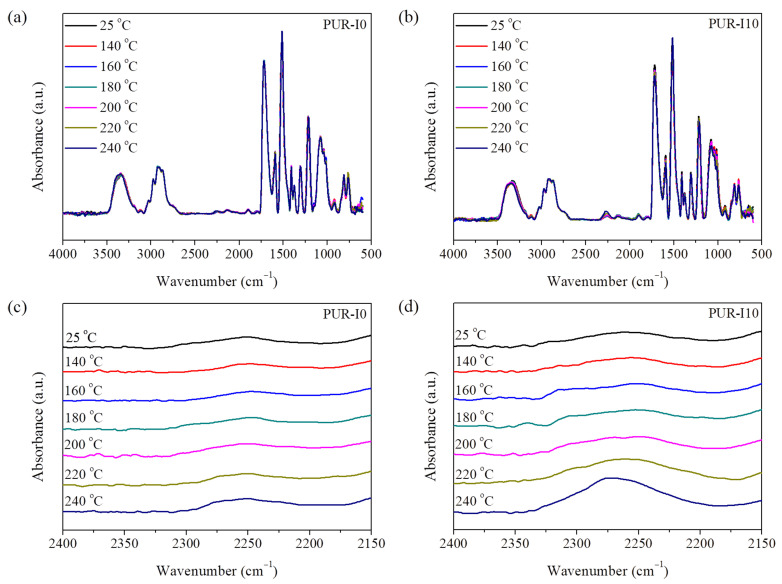
Temperature-dependent FTIR spectra of RPUFs: (**a**) PUR-I0; (**b**) PUR-I10; (**c**) PUR-I0 expanded at 2150–2400 ${\mathrm{cm}}^{-1}$; and (**d**) PUR-I10 expanded at 2150–2400 ${\mathrm{cm}}^{-1}$.

In previous studies, the reversible reaction of ISB-based urethane bonds was confirmed above 160 ${\,}^{\circ }C$ in flexible urethane foam and elastomer states. FTIR spectra were collected using the methods described in earlier research [51]. Figure 5 shows the temperature-dependent FTIR spectra of PUR-I0 and PUR-I10, normalized against the intensity of the C-H peak at 2966 ${\mathrm{cm}}^{-1}$. A weak peak intensity of isocyanate functional groups in RPUFs was observed between 2260 and 2280 ${\mathrm{cm}}^{-1}$ due to the use of excess isocyanate. As the temperature increased, PUR-I0 showed no change in the isocyanate peak, whereas PUR-I10 exhibited an increase in the isocyanate peak intensity above 200 ${\,}^{\circ }C$, which is higher than its $T_{g}$. This regeneration of isocyanate groups at relatively high temperatures can be attributed to the high cross-linking density and $T_{g}$ of RPUFs. These regenerated isocyanate groups, with restricted motion, could react with adjacent hydroxyl groups to regenerate urethane bonds.

**Figure 6:**
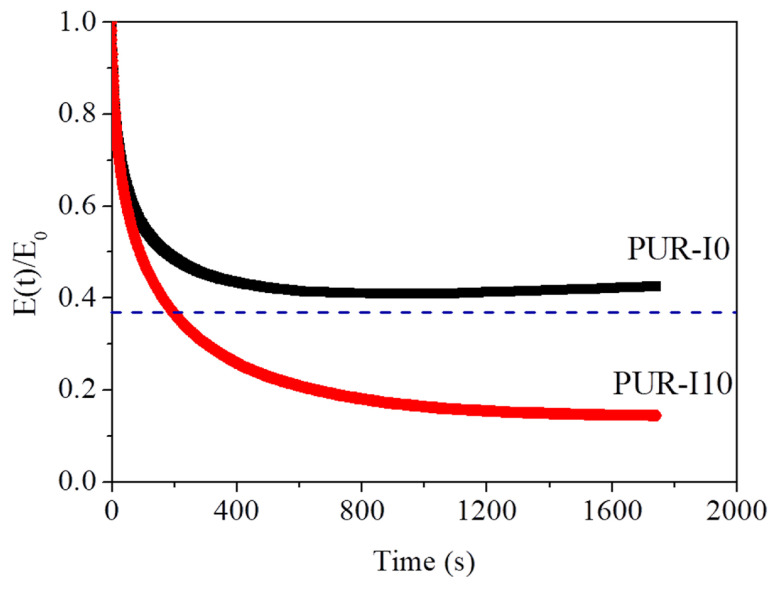
Normalized stress relaxation modulus (E(t)/E(0) of PUR-I0 and PUR-I10 at 160 ${\,}^{\circ }C$ at 1% strain (blue dotted line indicates 1/e (0.37) of the initial value).

In the stress relaxation test shown in Figure 6, PUR-I10 exhibited a faster decrease in normalized relaxation modulus compared to PUR-I0, reaching 1/e. This decrease in modulus might be due to the reversibility of the ISB-based urethane bonds. In other words, based on previous studies, it is assumed that ISB-based urethane bonds become reversible above 160 ${\,}^{\circ }C$ during the initial foaming process, where the cross-link density and polymer modulus are moderate. Generally, the maximum temperature of RPUFs during foaming can reach 160 ${\,}^{\circ }C$ due to the exothermic heat of reaction, which is sufficient to induce the reversibility of the ISB-based urethane bonds. Therefore, it is postulated that the reversible reaction of ISB-based urethane bonds during the initial foaming process could mitigate excessive cell growth or breakage caused by expansion and rupture. It is even anticipated that cells broken by foaming pressure may recover through the formation and dissociation of ISB-based urethane bonds. Consequently, smaller and more uniform cells, along with an increase in closed cell content, could be achieved, leading to a decrease in the thermal conductivity of ISB-based RPUFs.

**Figure 7:**
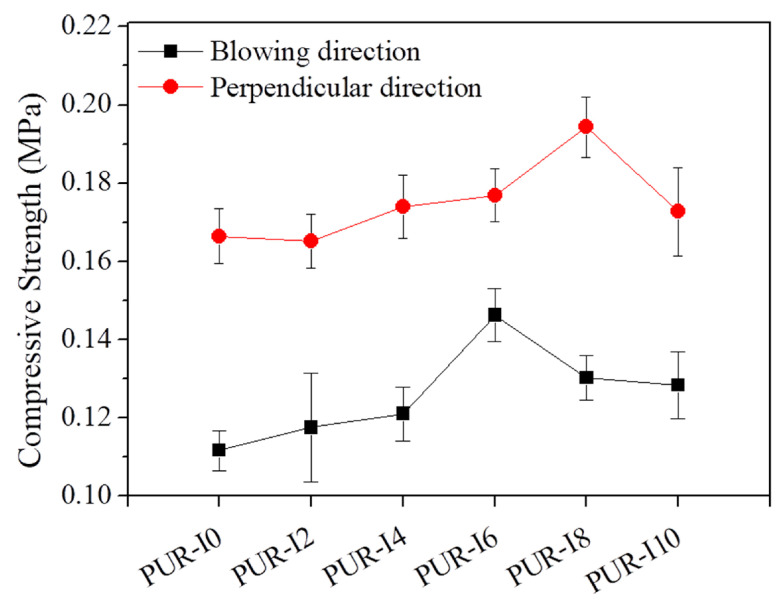
Compressive strengths in blowing direction (black squares) and perpendicular to the blowing direction (red circles) of RPUFs with different ISB contents.

Figure 7 shows the normalized compressive strength of RPUFs in both the blowing direction and the direction perpendicular to blowing, as well as the compressive strength at 10% deformation of the original height. The maximum compressive strength in the blowing direction and the direction perpendicular to blowing was obtained with PUR-I6 (0.15 MPa) and PUR-I8 (0.19 MPa), indicating 31% and 17% improvement, respectively. Generally, the RPUFs containing bio-based resources exhibit a decrease in mechanical properties caused by the reduced crosslinking density due to its high molecular weight and low functionality compared with conventional polyol for RPUF [15,16,17]. Incorporating ISB into RPUFs may loosen the cross-linked network by increasing the distance between cross-linking points, potentially deteriorating mechanical properties. Additionally, the reduction in average cell wall thickness could negatively impact compressive properties. However, the compressive strength in both measured directions did not weaken with the incorporation of ISB. On the contrary, ISB-based RPUFs demonstrated improved compressive strength compared to the control foam. This improvement is attributed to the smaller cell size and the rigid fused bicyclic structure of ISB. Therefore, incorporating ISB not only affects the mechanical properties, but also enhances the thermal properties of the RPUFs.

**Figure 8:**
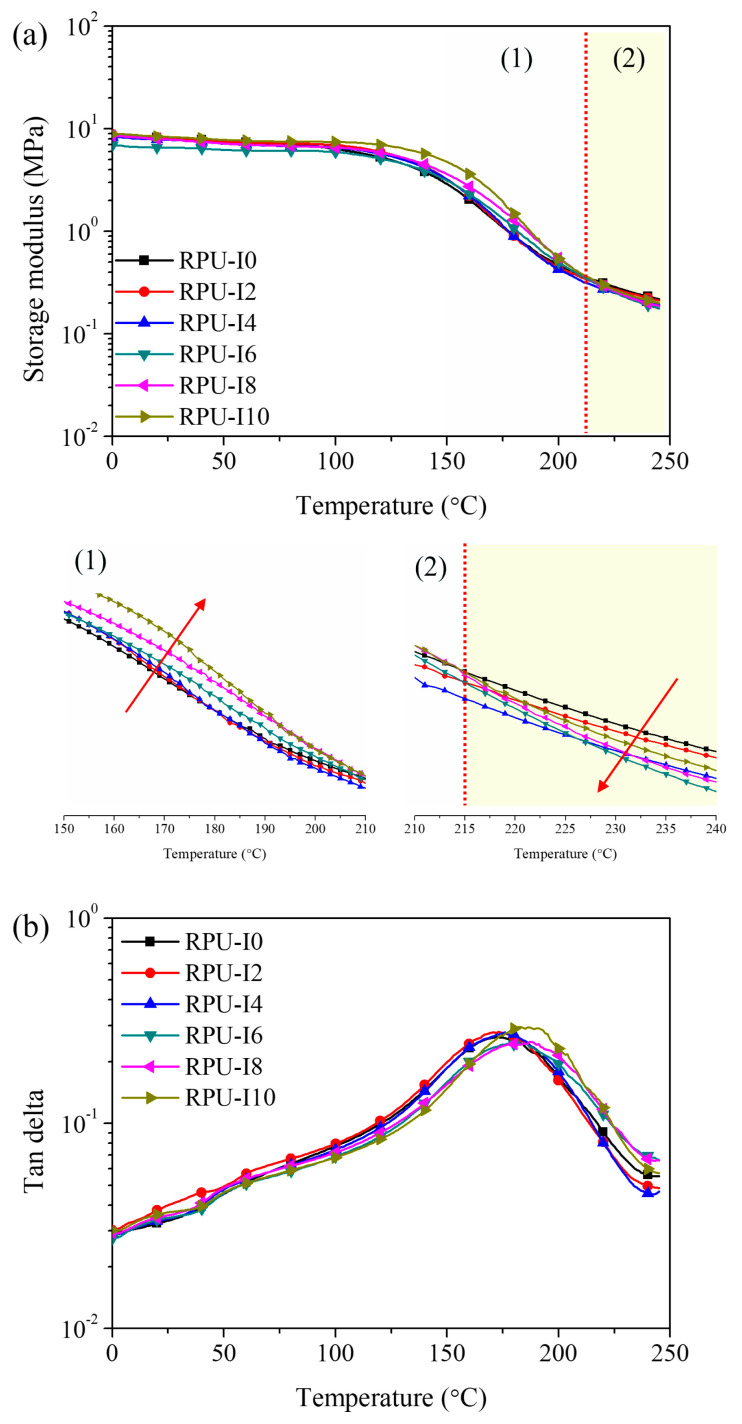
Storage modulus (**a**) and tan delta (**b**) curves of RPUFs with different ISB contents; (1) temperature range 150–210 ${\,}^{\circ }C$ and (2) temperature range 210–240 ${\,}^{\circ }C$ for the expanded region of (**a**).

Accordingly, the dynamic mechanical properties of RPUFs with varying ISB contents as a function of temperature are shown in Figure 8. Table 4 summarizes the glass transition temperature ($T_{g}$) data, determined from the maximum points of the tan delta curves. In the storage modulus curves, the moduli of RPUFs exhibited a plateau region, where the modulus remained constant up to the $T_{g}$. However, above the $T_{g}$, where molecular chains begin to vibrate or move, the moduli of RPUFs gradually decreased. In the transition region (130–210 ${\,}^{\circ }C$), the storage modulus of ISB-based RPUFs was higher than that of PUR-I0, but the moduli crossed at 215 ${\,}^{\circ }C$. Beyond this crossover point, the storage modulus of PUR-I0 exceeded that of ISB-based RPUFs, which could be attributed to the reversible nature of ISB-based urethane bonds. Additionally, the $T_{g}$ of ISB-based RPUFs increased with ISB content due to the restriction of molecular chain motion, resulting from the relative rigidity of ISB.

**Figure 9:**
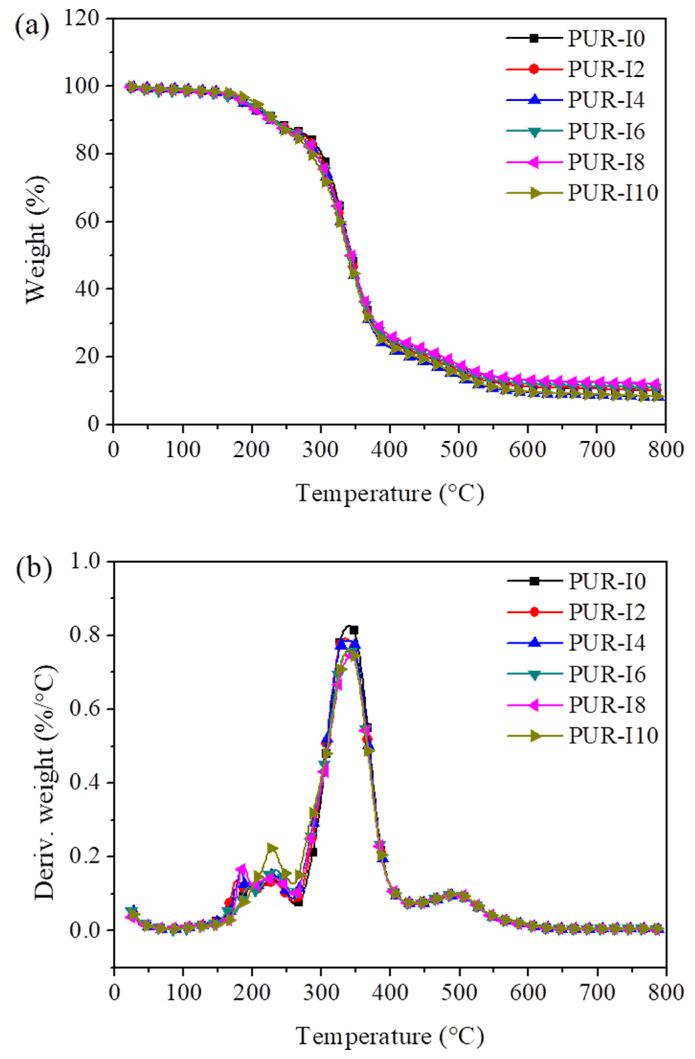
TG (**a**) and DTG (**b**) thermograms of RPUFs with different ISB contents in N_2_ atmosphere.

Figure 9 presents the TGA thermograms of RPUFs with varying ISB contents under a nitrogen atmosphere. The TGA curves for all RPUFs exhibited three distinct thermal degradation stages. The first stage, occurring between 170 ${\,}^{\circ }C$ and 250 ${\,}^{\circ }C$, corresponds to the dissociation and breakdown of urethane bonds. The second stage, between 270 ${\,}^{\circ }C$ and 430 ${\,}^{\circ }C$, is attributed to the thermal degradation of the polyol, while the third stage, observed between 430 ${\,}^{\circ }C$ and 540 ${\,}^{\circ }C$, corresponds to the thermal degradation of aromatic isocyanate and hydrocarbons [52,53,54]. Table 4 summarizes the maximum degradation temperatures ($T_{max}$) for each stage, as well as the thermal degradation temperatures at 5%, 10%, and 50% weight loss. The TG and DTG curves closely overlapped, indicating that the maximum degradation temperatures did not significantly change with varying ISB content. This suggests that incorporating ISB does not impact the thermal degradation behavior of RPUFs, and the thermal stability of ISB-based RPUFs is comparable to that of the control RPUF.

**Figure 10:**
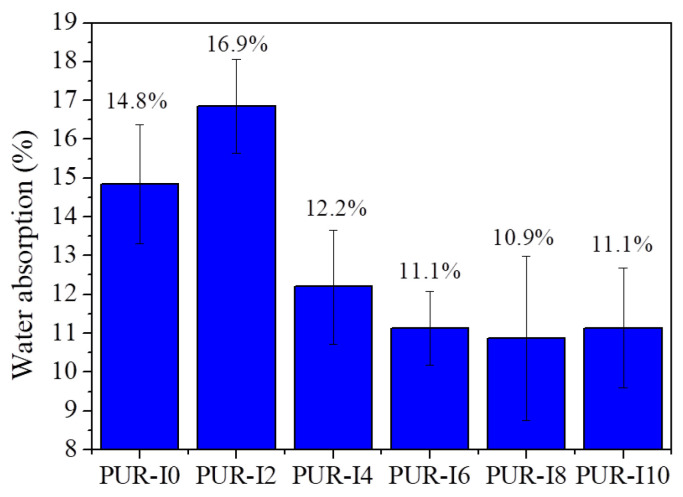
Percentage of water absorption by RPUFs with different ISB contents at room temperature after 24 h.

Figure 10 shows the percentage of water absorption by RPUFs with varying ISB contents. In practical applications, water or moisture absorption can lead to deformation, such as shrinkage or distortion, and deterioration of thermal insulation properties. Since ISB is hydrophilic due to the presence of oxygen atoms in its fused rings, this aspect was investigated. The water absorption test was conducted at room temperature for 24 h, with all surfaces and edges of the samples fully immersed in distilled water. The percentage of water absorption for PUR-I0 was 15.6%. A reduction in water absorption was observed as ISB content increased, with values of 13.7%, 10.2%, 9.8%, 9.7%, and 8.8% for PUR-I2, PUR-I4, PUR-I6, PUR-I8, and PUR-I10, respectively. This decrease in water absorption may be attributed to the steric hindrance effect of the V-shaped ISB in a highly cross-linked structure, making interaction with water molecules more difficult. Additionally, the increase in the closed cell content of ISB-based RPUFs likely contributed to the lower water absorption [1,55]. A significant reduction in water absorption was particularly noted for RPUFs with ISB content above 4 wt%, which coincided with a noticeable decrease in average cell size and an increase in the number of cells per unit volume [56].

## 4. Conclusions

In this study, different contents of isosorbide (ISB) were successfully incorporated into rigid polyurethane foams (RPUFs) and various polyurethane systems. ISB-based RPUFs exhibited improved thermal insulation and enhanced resistance to compression. ISB, a bio-based resource with a rigid fused bicyclic structure and two hydroxyl group, was dissolved in commercial polyether polyol at concentration up to 10 wt% for RPUFs production. The improved compatibility of ISB with conventional raw materials used in RPUF production played a key role in enhancing both thermal and mechanical properties. Notably, the ISB-based RPUFs did not exhibit shrinkage or deformation, despite a reduction in cross-linking density. Commercial RPUFs typically have a compressive strength ranging from 0.15 to 0.5 MPa and a thermal conductivity between 0.018 and 0.025 kcal/mh ${\,}^{\circ }C$. The incorporation of ISB resulted in smaller and more uniform cell sizes compared to the control foam (PUR-I0), leading to a 4% increase in closed cell content. This improvement contributed to the reduction in thermal conductivity, with the ISB-based RPUFs showing a decrease from 0.0192 to 0.0183 kcal/mh ${\,}^{\circ }C$, demonstrating enhanced thermal insulation properties. Additionally, compressive strength increased to 0.19 MPa, enhancing the material’s durability. The reversibility of ISB-based urethane bond also contributed to the enhancement of these properties. It is assumed that during the initial foaming process, the formation and dissociation of ISB-based urethane bonds, induced by reaction heat, contributed to controlling cell growth and reducing cell breakage. This mechanism is likely responsible for the observed decrease in cell size and increase in closed cell content, further reducing the thermal conductivity of ISB-based RPUFs.

In conclusion, the successful incorporation of ISB into various PU systems, particularly RPUFs, has demonstrated that ISB’s unique properties, including its rigid structure and reversible urethane bond formation, can enhance both thermal insulation and compressive strength. Notably, it exhibits excellent thermal insulation properties, making it a viable alternative for various applications, including building insulation materials, insulation for refrigerated and frozen containers, and household appliances—particularly as an insulating material for refrigerators. Further studies should focus on improving the miscibility between polyols and ISB, which could allow for a higher bio-content while maintaining desirable material properties. Also, investigating how dynamic reversibility of ISB-based urethane bonds at elevated temperature could provide valuable insights into the materials performance in RPUFs system. Future work should also explore the self-healing capabilities of ISB-based PU foams to further enhance the sustainability and performance of PU materials.

## Figures and Tables

**Table 1:** Sample codes and formulations of RPUFs with different ISB contents.

Components	PUR-I0	PUR-I2	PUR-I4	PUR-I6	PUR-I8	PUR-I10
	Composition by wt.					
Polyol part						
JOP-0655	100	98.0	96.0	94.0	92.0	90.0
ISB	0.0	2.0	4.0	6.0	8.0	10.0
TCPP	15.0	15.0	15.0	15.0	15.0	15.0
B-8462	2.0	2.0	2.0	2.0	2.0	2.0
PC-5	0.2	0.2	0.2	0.2	0.2	0.2
PC-8	2.4	2.4	2.4	2.4	2.4	2.4
365/227	30.0	30.0	30.0	30.0	30.0	30.0
H_2_O	1.0	1.0	1.0	1.0	1.0	1.0
Isocyanate part						
pMDI	142.7	144.6	146.6	148.6	150.5	152.5
NCO index	120	120	120	120	120	120

**Table 2:** Foaming process parameters and physical properties of RPUFs with different ISB contents.

Parameters	PUR-I0	PUR-I2	PUR-I4	PUR-I6	PUR-I8	PUR-I10
CT (s)	13	13	13	12	12	12
GT (s)	129	126	123	120	118	117
TFT (s)	187	178	175	170	169	166
Density (kg/m^3^)	46	45	46	45	45	46

**Table 3:** Morphological properties of RPUFs with various ISB contents.

Properties	PUR-I0	PUR-I2	PUR-I4	PUR-I6	PUR-I8	PUR-I10
Average cell size (μm)	475 ± 142	418 ± 84	394 ± 78	354 ± 63	294 ± 54	302 ± 49
Average thickness of cell walls (μm)	33.1 ± 9.8	30.9 ± 6.2	25.8 ± 7.3	26.9 ± 8.5	24.8 ± 8.5	22.6 ± 6.7
Number of cells ($10^{4}$ cells/cm^3^)	1.82	2.37	3.70	3.95	4.58	4.96

**Table 4:** Characteristic decomposition temperatures and $T_{g}$ of various RPUFs.

Decomposition Temperature (${\,}^{\circ }C$)	PUR-I0	PUR-I2	PUR-I4	PUR-I6	PUR-I8	PUR-I10
$T_{g}$ ^1^	173.0	174.0	176.9	182.6	187.9	187.0
$T_{5\%}$ ^2^	194.4	187.3	187.3	194.4	190.5	204.0
$T_{10\%}$ ^3^	232.9	229.7	225.2	232.9	227.1	231.0
$T_{50\%}$ ^4^	344.8	342.6	340.0	342.6	343.9	340.0
$T_{max1}$ ^5^	229.7	230.3	231.6	232.2	230.3	227.7
$T_{max2}$ ^6^	340.7	335.5	339.4	340.0	342.6	340.0
$T_{max3}$ ^7^	494.6	494.0	491.4	492.7	494.6	495.3

^1^ $T_{g}$ determined by the maximum point of the tan delta curve obtained from DMA measurement; ^2^ $T_{5\%}$ indicates the decomposition temperature at 5 wt% of weight loss; ^3^ $T_{10\%}$ indicates the decomposition temperature at 10 wt% of weight loss; ^4^ $T_{50\%}$ indicates the decomposition temperature at 50 wt% of weight loss; ^5^ $T_{max1}$ indicates the maximum decomposition temperature of the first decomposition stage; ^6^ $T_{max2}$ indicates the maximum decomposition temperature of the second decomposition stage; ^7^ $T_{max3}$ indicates the maximum decomposition temperature of the third decomposition stage.

## Data Availability

The data presented in this study are available within this article.
